# Population growth of *Varroadestructor* (Acari: Varroidae) in commercial honey bee colonies treated with beta plant acids

**DOI:** 10.1007/s10493-014-9821-z

**Published:** 2014-05-15

**Authors:** Gloria DeGrandi-Hoffman, Fabiana Ahumada, Robert Curry, Gene Probasco, Lloyd Schantz

**Affiliations:** 1 Carl Hayden Bee Research Center, USDA-ARS, 2000 East Allen Road, Tucson, AZ 85719 USA; 2 AgScience Consulting LLC, Tucson, AZ USA; 3 Crystal River Consulting, Dallas, TX USA; 4 BetaTec Hop Products, Division of John I. Haas Inc., 5185 MacArthur Blvd, N1 V, Suite 300, Washington, DC 20016 USA

**Keywords:** Migration, Population dynamics, Dispersal, *Apismellifera*, Parasite

## Abstract

Varroa (*Varroa destuctor* Anderson and Trueman) populations in honey bee (*Apis* *mellifera* L.) colonies might be kept at low levels by well-timed miticide applications. HopGuard^®^ (HG) that contains beta plant acids as the active ingredient was used to reduce mite populations. Schedules for applications of the miticide that could maintain low mite levels were tested in hives started from either package bees or splits of larger colonies. The schedules were developed based on defined parameters for efficacy of the miticide and predictions of varroa population growth generated from a mathematical model of honey bee colony–varroa population dynamics. Colonies started from package bees and treated with HG in the package only or with subsequent HG treatments in the summer had 1.2–2.1 mites per 100 bees in August. Untreated controls averaged significantly more mites than treated colonies (3.3 mites per 100 bees). By October, mite populations ranged from 6.3 to 15.0 mites per 100 bees with the lowest mite numbers in colonies treated with HG in August. HG applications in colonies started from splits in April reduced mite populations to 0.12 mites per 100 bees. In September, the treated colonies had significantly fewer mites than the untreated controls. Subsequent HG applications in September that lasted for 3 weeks reduced mite populations to levels in November that were significantly lower than in colonies that were untreated or had an HG treatment that lasted for 1 week. The model accurately predicted colony population growth and varroa levels until the fall when varroa populations measured in colonies established from package bees or splits were much greater than predicted. Possible explanations for the differences between actual and predicted mite populations are discussed.

## Introduction

Varroa mites (*Varroa destructor* Anderson and Trueman) are the most serious pest of honey bee (*Apis mellifera* L.) colonies worldwide (Rosenkranz et al. 2010). Varroa is an ectoparasite that feeds on developing brood and adults. Colony losses from varroa are due to brood mortality and the reduction in the lifespan of adult workers that were parasitized during development (Rosenkranz et al. 2010). The combination of reduced rates of brood emergence and short-lived adults impact the demographics of the colony population and over time can cause colonies to perish (DeGrandi-Hoffman and Curry 2004; van Dooremalen et al. 2012). Varroa also transmit many types of virus during feeding (Ball and Allen 1988; Bowen-Walker and Gunn 1998; Bowen-Walker et al. 1999; Chen et al. 2004; Shen et al. 2005; Di Prisco et al. 2011). Varroa mediated transmission of deformed wing virus is a major cause of colony losses overwinter (Guzman-Novoa et al. 2010).

Varroa populations increase as the broodnest of a colony expands in the spring and summer. Mated female mites (foundress) invade brood cells just before they are capped for pupation (see refs in Rosenkranz et al. 2010). Male and female offspring are produced and mate under the sealed cell. Each invading ‘mother mite’ can produce about 1.5 mated female offspring in worker cells and 2.7 in drones (Fries et al. 1994; Martin 1998). The mated female mites leave the cell when the bee emerges and in this phoretic stage search for new cells to infest. It is during the phoretic stage that the mite is most vulnerable to chemical control measures.

The reproductive rate of varroa is not extraordinarily high. If colonies are established with low mite levels in temperate climates, it takes 2–3 years before mite populations are large enough to impact the colony’s survival (Boecking and Genersch 2008; Genersch 2010; Rosenkranz et al. 2010). In addition to reproduction, mite populations can increase due to the drifting of foragers from infested colonies or robbing failing colonies infested with mites (Sakofski et al. 1990; Greatti et al. 1992; Kralj and Fuchs 2006). The extent that immigration affects mite population growth in colonies is not known.

Varroa control should be based on well-timed treatments that keep varroa populations low and their growth rates in check. Mathematical models can provide tools for developing strategies to optimize the timing of miticide applications to maximize their impact on mite populations. There are several mathematical models simulating honey bee colony and varroa population dynamics (Fries et al. 1994; Martin 1998, 2001; Calis et al. 1999; Wilkinson and Smith 2002; DeGrandi-Hoffman and Curry 2004; Vetharaniam 2012; see refs. in Becher et al. 2013). We used the model developed by DeGrandi-Hoffman and Curry (2004) to develop mite control strategies. The model generates daily predictions of colony population size (adults and brood) and numbers of phoretic mites so that field data could be compared with model predictions. Additionally, we could initialize simulations with numbers of adult bees, brood, and mite populations using data collected at our apiary sites. The model also simulates mite mortality from the application of miticides. The efficacy, dates of application and period of effectiveness of the miticide are specified as initial conditions in the simulation.

The model predicts that in temperate climates it could take up to 2 years for mite populations to reach levels where they cause colony losses if initial populations are below 1.0 mites per 100 bees in the spring. These predictions are similar to reports of colonies dying after 2–3 years of untreated varroa infestation (Rosenkranz et al. 2010). The model also predicted that late summer treatments could reduce mite populations so that the bees that comprise the winter cluster are reared with minimal exposure to parasitism. This agrees with findings from field studies by Delaplane and Hood (1999).

We used the model to develop treatment schedules to control mites in colonies with HopGuard^®^ (HG; BetaTec Hop Products, Washington, DC, USA). The active ingredient in this product is beta plant acids. We chose HG because it can be applied to package bees and colonies during the summer and early fall when temperatures are high. Our previous studies determined the efficacy of HG in package bees and established colonies (DeGrandi-Hoffman et al. 2012). In the present study, we established colonies in the spring from package bees or splits from larger colonies and treated them with HG. The purpose of this study was to determine whether varroa can be controlled in commercial colonies using beta plant acids (e.g., HG) by reducing mite levels after establishment and following this with well-timed applications of HG.

## Materials and methods

### Varroa treatments in colonies started from packages

Bee packages weighing 1.4 kg and containing approximately 9,000 bees were prepared at Pendell Apiaries (Stonyford, CA, USA) on May 2, 2011. Twenty packages were treated with HG for 48 h before installation into the hives. Five packages received no treatment (controls). Packages were treated by fastening a HG strip to the top of the package near the sugar syrup can and caged queen. All packages were kept inside a climate-controlled room. After 48 h and at dusk, each bee package was installed in a standard deep Langstroth hive box fitted with foundation frames and a queen. Thereafter, the bees were fed sugar syrup dispensed by a 4-l (= 1-gallon) can inverted in the top lid. The apiary site had only the colonies used in this study. The closest colonies not included in the study were 2.4–3.2 km away.

On May 12, the initial mite population in each colony was estimated using the ‘sugar shake’ method. Approximately 300 bees were brushed into mason jars with wire screen lids. Powdered sugar was added to each jar through the wire screen. Each jar was rolled gently to cover the bees and then set aside for 2–3 min. The jars were then inverted and shaken vigorously over a wide aluminum foil pan filled with 2.5 cm of water until there was no sugar left in the jars. The mites were counted in the pan, and the bees were placed back in each colony. The mite counts were converted to ‘mites per 100 bees’. The colonies were assigned into groups of five hives based on their mite levels. Each group was assigned an HG treatment schedule (Table 1). Untreated control colonies comprised group 5.

**Table 1:** Treatment groups based on times when the miticide HopGuard^®^ (HG) was applied to control varroa mites

Group	Package	June 22	August 4	October 11
1	X	X		
2	X	X	X	X
3	X		X	X
4	X			
5 (control)				

The colonies were started from packages, and HG was applied to the bees in the package. Subsequent treatments in colonies were applied on the dates indicated. Controls received no HG treatments

Treatment schedules for colonies established from packages were devised based on simulation results using a varroa–honey bee colony population dynamics model (DeGrandi-Hoffman and Curry 2004). The simulations identified times when HG applications could be applied to keep varroa populations below levels where they might cause colony losses. Various numbers of treatments throughout the summer and fall were included in the schedules to determine the minimum number required for effective control.

Each treatment consisted of two HG strips inserted into each 10-frame hive following label instructions. Mite levels were measured in all colonies before and 48 h after an HG treatment. The post-treatment interval was chosen because the greatest portion of mites that are killed by HG occur 48 h after application (DeGrandi-Hoffman et al. 2012). In addition to estimating mite populations, frames of adult bees and brood were measured prior to HG treatment in all colonies using the methods described in DeGrandi-Hoffman et al. (2008). Colony estimates were made in August and October. The presence of a laying queen also was recorded at these times.

### Varroa treatments in colonies made from splits

Full sized colonies at the Adee Honey Farms were split into smaller colonies on March 24, 2012. These colonies are hereafter referred to as ‘splits’. A sealed queen cell was added to each split. The splits were located in an apiary near Fresno (CA, USA) that contained about 1,800 hives not included in this study. Those colonies also were treated for varroa; 900 colonies with HG and the remainder with another miticide.

Colony population sizes were estimated (frames of adult bees and brood, presence of laying queen) 19 days after the splits were made (i.e., April 11, 2012). There was little or no sealed brood in the colonies at this time because brood from the new queen would not be sealed, and most of the unsealed brood in the split would have developed and emerged. Any remaining sealed brood would have emerged within 48 h, and this would be during the effective period for the HG treatment. Simulations predicted that HG treatments during the broodless period could reduce mite populations to <1.0 mite per 100 bees, and that the populations would remain low during the experimental interval. Therefore, we chose this time for initial HG applications.

Twenty-four colonies selected for treatment hives averaged 6.3 ± 0.3 frames of adult bees, and 2.5 ± 0.3 frames of brood. The colonies were treated with two HG strips inserted between the center frames of the broodnest. Similarly, 24 colonies were selected as control hives. These colonies averaged 6.4 ± 0.2 frames of adult bees and 2.8 ± 0.3 frames of brood. The 24 control colonies received no miticide treatment. Estimates of mite populations in all colonies were made immediately before and 48 h after treatment using the procedure described above for the package colonies.

All colonies (those in the study and the other 1,800 hives) were moved to Adee apiary sites in South Dakota for the summer, and returned to isolated yards in Arvin, CA, in September. We were not aware of any colonies other than those belonging to the Adee’s at either apiary site. Colony strength and mite levels were estimated on September 11, 2012 using the procedures described above. At this time, the 24 treatment colonies were divided into two groups of 12 colonies. One group received an HG treatment (as described above) on September 12. A second group of 12 colonies received weekly HG treatments for 3 consecutive weeks beginning on September 12. The control colonies also were divided into two groups of 12 colonies each. Twelve control colonies received no HG treatments. The remaining 12 colonies received three consecutive HG treatments applied 1 week apart. Pre- and post-treatment mite counts were made using the methods described above. This experimental design enabled us to compare the effectiveness of single HG treatments with those that lasted for an entire worker brood cycle in colonies having open and sealed brood and different mite population levels. Final measurements of mites per 100 bees, and frames of bees and brood were made on November 12 using the procedures described above.

### Conditions common to all varroa-colony population dynamics model simulations

The model used to predict varroa and colony population sizes is described fully in DeGrandi-Hoffman and Curry (2004). The model generates daily predictions of colony size (adults and immature), phoretic mites and infested worker and drone cells. The predictions are based on initial colony size, queen egg laying potential (queen strength), worker longevity, initial mite infestation levels and weather conditions. The weather conditions used in the simulations were chosen based on the areas and times of year when the experiment was conducted.

Queen strength is defined in the model as the maximum number of eggs a queen potentially can lay in a day and was initialized in simulations as 1,000–2,000 eggs unless noted otherwise. The range was captured by conducting separate simulations for each set of initial conditions (colony and mite population sizes) where queen strength was initialized as either 1,000, 1,500 or 2,000 eggs per day.

Measurements of bees and brood in the field were expressed as frames covered with adults or with open and sealed brood. The model expresses colony populations with daily estimates of: adult bees, sealed and unsealed worker and drone brood, and eggs (worker and drone). We converted colony population estimates from the model into total number of adult bees and frames of brood before comparing actual and predicted values. According to our measurements, the number of bees covering both sides of a deep frame was estimated to be 2,506 adults (data not shown). Therefore, we multiplied frames of adult bees in the actual colonies by 2,506 to estimate the total number of adult bees. Predicted totals of brood (sealed and unsealed) were converted to frames of brood by dividing the totals by our estimate of 5,200 cells on a deep frame with about 80 % containing brood.

Mortality of phoretic mites from HG treatments was included in each simulation beginning on the day that HG was applied. The model simulated a daily mortality rate of 50 % of phoretic mites for 7 days for each HG application (DeGrandi-Hoffman et al. 2012).

### Simulations: colonies established from packages

To simulate a colony started from a 1.4 kg package of bees, we initialized the model with 9,000 adult bees and no brood on May 12. Separate simulations were run to represent each colony in every group having the range of queen strengths described above. The colony specific estimate of the pre-treatment number of mites per 100 bees sampled on May 12 was used as the initial mite population. Thus, 15 simulations were run to obtain predictions of average mite populations and colony sizes for each group. The predicted averages of mites per 100 bees and colony population size were compared with actual data collected on the same date. The dates for HG treatments in the simulations were the same as in the actual colonies.

### Simulations: colonies established from splits

Colonies started from splits of larger colonies began with an average of 6.3 ± 0.3 deep frames of bees (15,787 ± 752 adults) and 2.8 ± 0.3 deep frames of brood. We simulated this range of colony populations by conducting separate simulations using upper and lower ranges of the standard error for adult bees and frames of brood. The range of maximum queen egg laying rates and worker longevity described above were included in simulations for each colony size. However, the model’s predictions of adult populations and frames of brood in September and November were much higher than actually occurred. When we adjusted the maximum queen egg laying rate to 1,500 eggs per day and worker longevity to 26 days, predictions of colony and brood population sizes were similar to the actual colonies. These parameter values were used for simulations of both treatment and control colonies. Our measurement of the initial mites per 100 bees in the treatment colonies was 2.5 ± 0.5 and controls was 1.2 ± 0.24 mites per 100 bees. These values were used as the starting infestation levels in the simulations. Predicted averages of frames of adult bees or brood and mites per 100 bees were estimated using values from four simulations each for treatments and controls.

### Statistical analysis

The average number of mites per 100 bees in colonies started from packages was compared among the groups immediately after the colonies were established and following HG treatments to any group using a one-way analysis of variance. A repeated measures analysis was conducted to determine if mite numbers differed among the sampling intervals. The effectiveness of the HG treatment 48 h after application was determined by comparing mite counts before and after treatments within each group using t tests.

Mite counts in colonies made from splits were compared between treatment and controls prior to and after HG treatments using t tests. Mite levels among treatment and control colonies receiving one, three, or no HG treatments in September were compared using a one-way analysis of variance. The accuracy of predictions from the model were assessed by comparing actual and predicted averages of mites per 100 bees, adult bees in colonies and frames of brood using t tests.

## Results

### Varroa mortality in colonies established from packages

**Fig. 1:**
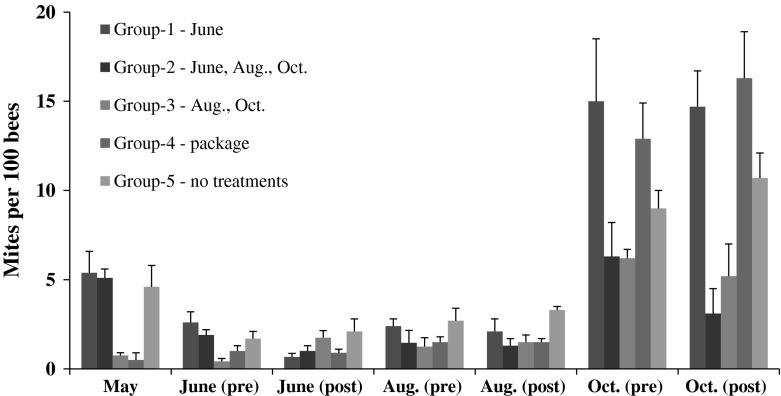
Mites per 100 bees estimated from sugar shakes of colonies started from package bees and treated with the miticide HopGuard^®^ (HG). All colonies except those in group 5 (Control) were treated in the package prior to establishing them in colonies. Months when subsequent HG treatments were made for the group are defined in the figure legend

Groups of colonies had significantly different numbers of mites per 100 bees in May immediately after they were established from packages (F_4,18_ = 7.16, *p* = 0.001) (Fig. 1). Colonies in groups 1, 2 and 5 had the most mites and groups 3 and 4 the least. After groups 1 and 2 were treated in June, post-treatment mite levels were reduced and no longer significantly different from the other groups (June, pre-treatment: F_4,18_ = 3.64, *p* = 0.024; post-treatment: F_4,18_ = 4.19, *p* = 0.11). Colonies in groups 1–4 had less than 1.0 mite per 100 bees in June. Group 5 colonies (untreated controls) averaged 2.1 ± 0.67 mites per 100 bees.

In August, mite numbers did not differ among the five groups prior to HG treatments (F_4,18_ = 1.37, *p* = 0.28). After treatment though, colonies in groups where HG was applied (groups 2 and 3) had significantly fewer mites than group 5 (F_4,18_ = 3.57, *p* = 0.026). Mites per 100 bees in groups 1–4 averaged between 1.3 and 2.0 mites per 100 bees. Group 5 averaged about 3.3 ± 0.24 mites per 100 bees.

Prior to HG treatments in October, mite numbers were 3–7× higher than in August in all groups (Fig. 1). Repeated measures analysis indicated that mite numbers in all groups were significantly higher in October than during any other sampling interval (F_8,25_ = 28.9, *p* < 0.0001). The lowest mite numbers before the October treatments were in groups 2 and 3 that were previously treated in August (pre-treatment: F_4,18_ = 3.33, *p* = 0.03). These groups also had the lowest numbers of mites after the October treatment (F_4,18_ = 9.57, *p* < 0.0001).

Comparisons of mite counts in groups 1, 2 and 3 before and 48 h after HG applications (pre- vs. post-treatment) were significantly different for the June treatment in group 2 (t_5_ = 4.22, *p* = 0.013) and the October treatment in groups 2 and 3 (Table 2). There was no difference between pre- and post-treatment mite counts in groups receiving August treatments (groups 2 and 3). However, these treatments might have been effective at reducing mite populations since those colonies had the lowest mite numbers in October.

**Table 2:** Comparisons of average number of varroa mites per 100 bees before (pre-) and after (post-) treatment with the miticide HopGuard^®^

Group	Treatment month	Pre-treatment	Post-treatment	t	*p*
1	June	2.6 ± 0.6	0.7 ± 0.2	2.46	0.07
2	June	1.9 ± 0.3	1.0 ± 0.3	4.20	0.01
	August	1.5 ± 0.7	1.3 ± 0.8	0.23	0.83
	October	6.3 ± 1.9	0.5 ± 0.3	2.22	0.09
3	August	1.2 ± 0.5	1.5 ± 0.4	0.43	0.70
	October	2.9 ± 0.2	0.2 ± 0.1	8.69	0.01

There were five colonies in each group

### Varroa mortality in colonies established from splits

**Fig. 2:**
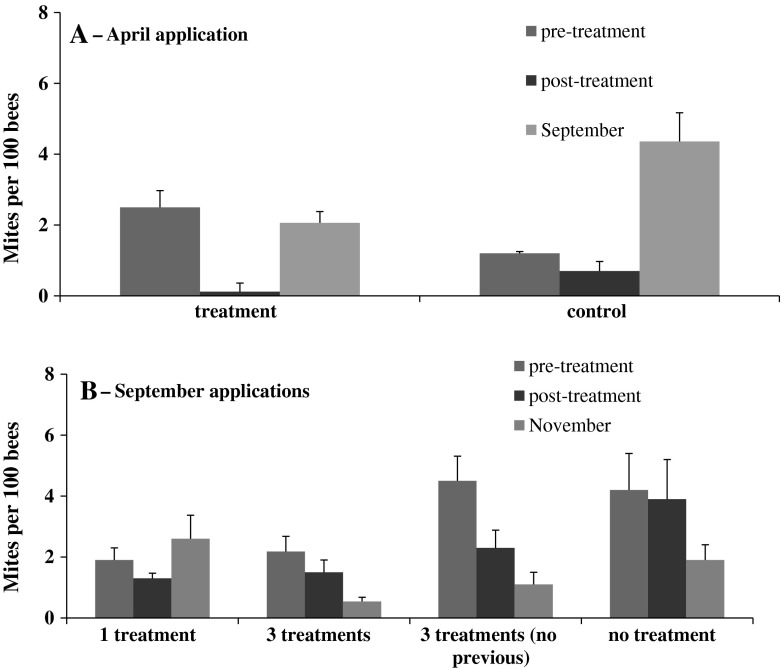
Mites per 100 bees in colonies pre- and post-treatment with the miticide HopGuard^®^, in April (**a**) and in September (**b**)

There were significantly more mites in treatment colonies than in controls prior to HG applications in April (treatment = 2.5 ± 0.47 mites per 100 bees, controls = 1.2 ± 0.24 mites per 100 bees; t_34_ = 2.34, *p* = 0.025) (Fig. 2). After the HG application, however, treatment colonies had significantly fewer mites than controls (treatment = 0.12 ± 0.05 mites per 100 bees; controls = 0.7 ± 0.27 mites per 100 bees; t_24_ = 2.13, *p* = 0.043).

Before treatments in September, the colonies treated in April still had significantly fewer mites (1.3 ± 0.25 mite per 100 bees) than untreated controls (4.2 ± 1.2 mites per 100 bees) (t_26_ = 2.64, *p* = 0.014).

HG was applied in September to all treatment colonies and half of the control colonies. Those colonies that had three consecutive HG treatments in September had significantly fewer mites per 100 bees in November than colonies that had one HG application or the controls that had no HG treatments (F_3,31_ = 3.56, *p* = 0.025). Mite numbers in colonies that had one HG application in September did not differ from the untreated controls.

### Comparisons of actual and predicted mite and colony population growth

**Fig. 3:**
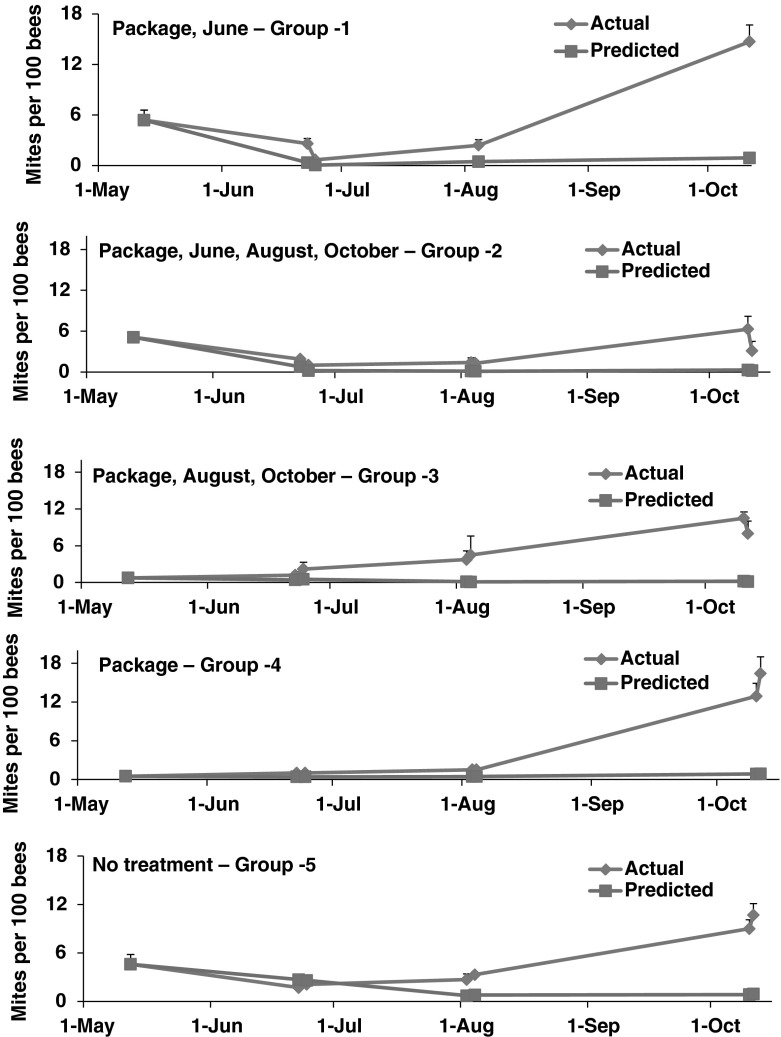
Actual and predicted averages of mites per 100 adult bees in colonies started from packaged bees on May (5 colonies per treatment Group). Packages were treated with the miticide, HopGuard ^®^ (HG). Colonies were treated subsequently with HG during the months designated in each plot. Actual and predicted mite numbers did not differ significantly during May, June, or August. However, in October actual mite numbers were significantly higher than predicted for all treatment schedules as determined by two sample t-tests (*p* < 0.05)

Predictions from simulations of mite populations in colonies started from packages were similar to those estimated in actual colonies in June and August (<0.5 mites per 100 bees) (Fig. 3). Untreated controls were predicted to have about 1.0 mite per 100 bees and this also was comparable to estimates in actual colonies. Additional treatments in August were predicted to keep mite populations low (<0.15 mites per 100 bees). Colonies treated in both June and August or in August alone did not differ in mite numbers before or after treatments, and during these intervals predicted mite populations were similar to actual counts for all treatment groups. However, actual mite populations in all treatment groups in October was 2.5- to 10-fold higher than model predictions.

**Fig. 4:**
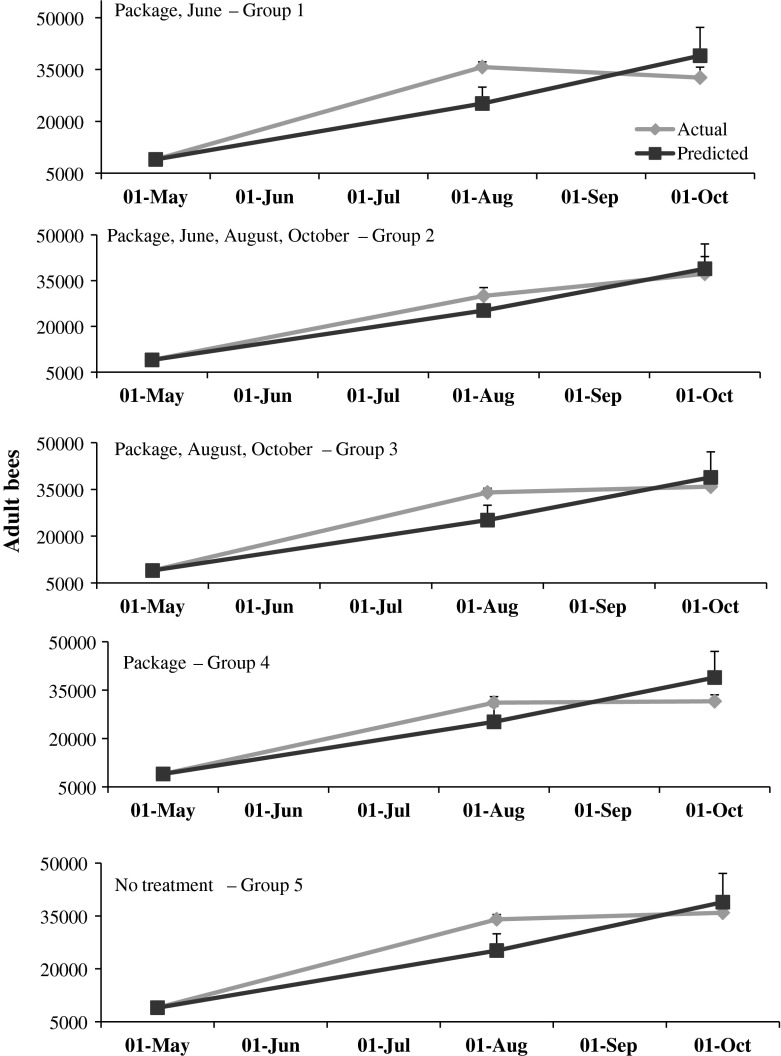
Actual and predicted numbers of adult bees in honey bee colonies started from package bees in May. Packages in groups 1–4 were treated with the miticide HopGuard^®^ (HG). Additional HG applications were made in colonies in groups 1, 2 and 3 during the months designated in each plot

**Fig. 5:**
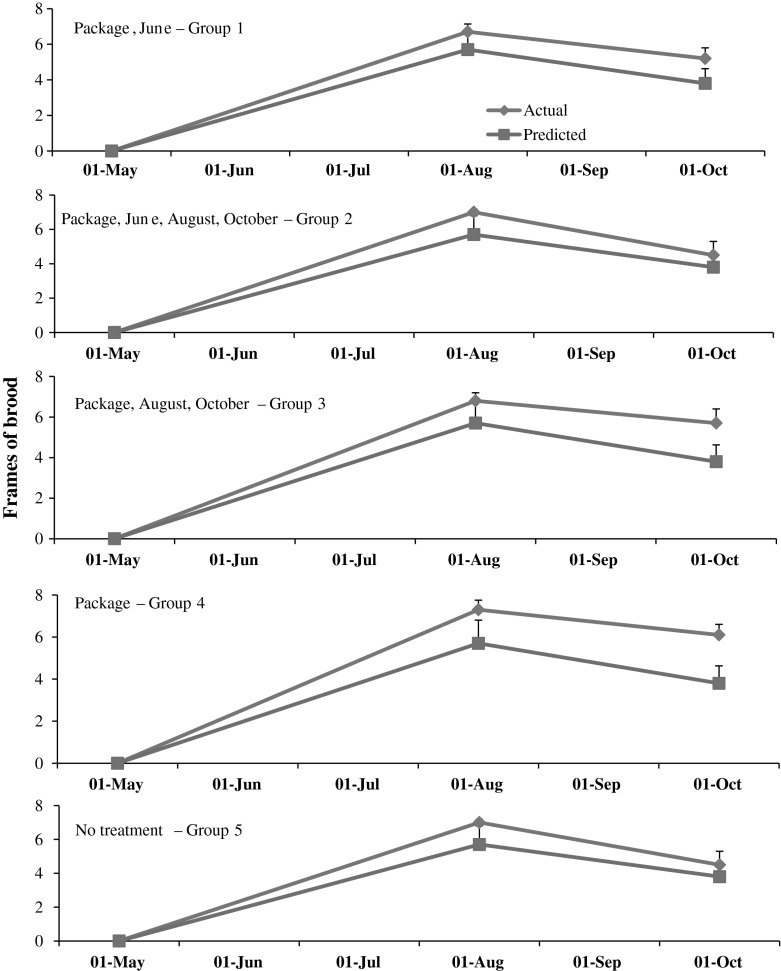
Actual and predicted frames of brood in honey bee colonies started from package bees in May. Packages in groups 1–4 were treated with the miticide HopGuard^®^ (HG). Additional HG applications were made in colonies in groups 1, 2 and 3 during the months designated in each plot

The model accurately predicted colony population growth following the establishment from packages. Based on t tests comparing actual and predicted averages, the predicted population sizes did not differ from the actual (*p* > 0.05) during any sampling interval (Fig. 4). Frames of brood also were similar between actual and predicted (*p* > 0.05) (Fig. 5).

**Fig. 6:**
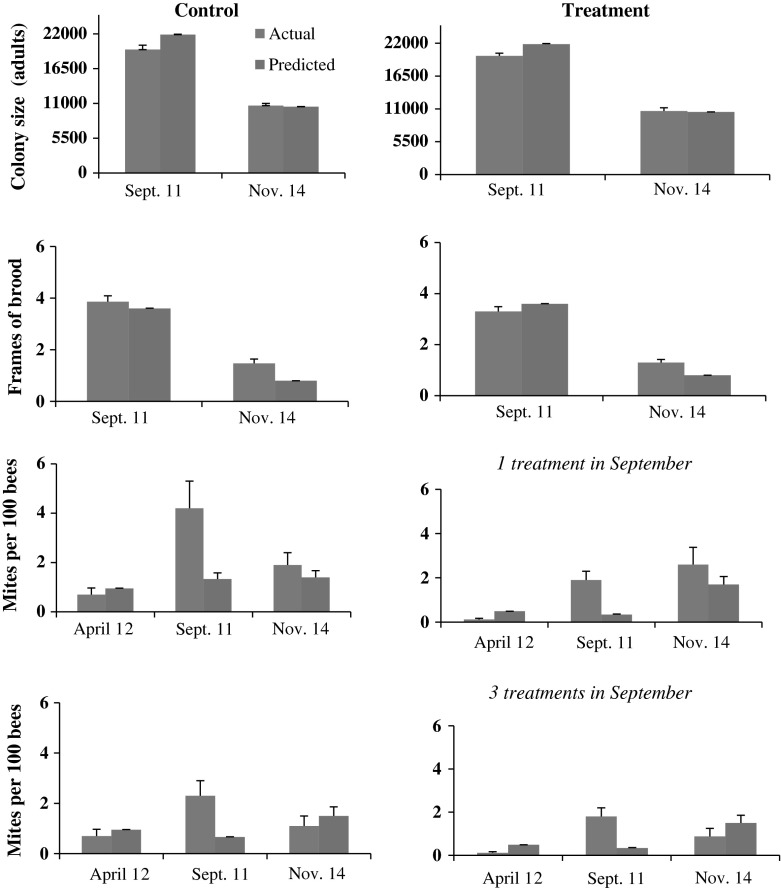
Actual and predicted colony and mite populations in hives established in March and treated with the miticide HopGuard^®^ (HG) in April (1 treatment) and September (1 or 3 treatments). Control colonies were either not treated or were treated only in September when they received 3 HG treatments

In colonies established from splits, the adjustments in queen strength and worker longevity generated predicted colony populations and frames of brood that were similar to those in the actual colonies (Fig. 6). There was no significant difference between actual and predicted mites per 100 bees following the April HG treatment (t_23_ = 7.14, *p* < 0.0001). Mite numbers in treatment colonies were predicted to be less than 0.5 mites per 100 bees. Estimates of the actual mite population in September before treatment, however, were about 12× higher than predicted in treatment colonies (actual = 1.8 ± 0.4 mites per 100 bees; predicted = 0.14 ± 0.01 mites per 100 bees). In control colonies, the actual number of mites per 100 bees was about 6× higher than predicted. In both instances, these differences between actual and predicted mite numbers were significant (treatment: t_21_ = 5.3, *p* < 0.0001; control: t_20_ = 3.73, *p* = 0.001). In November, predicted mite populations did not differ from actual in treatment colonies that received either one or three HG treatments (1 treatment: t_9_ = 0.98, *p* = 0.35; 3 treatments: t_6_ = 2.52, *p* = 0.05). Predicted mite counts in control colonies also did not differ from actual counts in November (no treatment: t_12_ = 0.6, *p* = 0.55; 3 treatments: t_12_ = 0.73, *p* = 0.48).

## Discussion

Mite populations were reduced when HG was applied in colonies started from package bees (summer treatments) and during the broodless period in colonies made from splits. Mite numbers did not exceed 2 mites per 100 bees until the fall. However, at the sites and sets of conditions in this study, mite populations in the fall were higher than predicted. Mite numbers could be reduced at these times especially if three consecutive HG applications were made. Mite populations at the study sites did not appear to be the product of mite reproduction alone especially by late summer and fall. Instead, increases in mite numbers might have been due to other factors such as the drifting of workers with phoretic mites from other colonies. The frequency of this activity might be higher than previously suspected and significantly increase mite populations before the colonies go into winter.

The period of effectiveness for HG is about 7 days, so mites in brood cells can emerge after the active ingredient in HG is no longer present (DeGrandi-Hoffman et al. 2012). Single HG treatments were effective in package bees or colonies without sealed brood because the mite population was composed of only those in the phoretic state. Significant mite reductions with a single HG application occurred in colonies with open and sealed brood in June and October but not in August when brood areas were at their peak. In the colonies started from splits, mite populations in November were no different from untreated colonies if they had only a single HG application in September. To improve the likelihood of reducing mite populations in colonies with sealed brood, three consecutive HG treatments should be applied so that the active ingredient is present for an entire brood cycle.

Though HG reduced mite populations in packages and split colonies during the spring and summer, by the fall mite numbers were many times higher than predicted by our model. The model predicts drone and worker brood population sizes and the probabilities of mites infesting them (mites are more likely to infest drone rather than worker brood). The reproductive rates of mother mites in worker and drone cells used in the simulations were based on reported values (Fries et al. 1994; Martin 1998) as were the percentages of mother mites that successfully reproduce after entering the cell (Martin 1994, 1995a, b; Rosenkranz and Engels 1994). The proportion of foundress mites that emerge and have additional reproductive cycles is considered in the model as are changes in mite reproductive rates if cells are multiply infested (see DeGrandi-Hoffman and Curry 2004). We based the initial conditions for the simulations on the actual colony and mites population sizes. The model generated predictions of mite populations that were similar to those measured in the field throughout the summer in colonies started from packages. Predictions of mite populations in colonies started from splits also were comparable to actual measurements following the April HG treatments. Predicted colony populations and frames of brood, which are the foundation for mite population growth, were similar to those measured in the field. Therefore, the increases in mite populations measured in colonies in the fall were due to factors not considered in the simulations or from causes other than mite reproduction.

The predictions of mite populations from our model are similar to reports that colonies established with low mite populations can survive for several years if untreated (Büchler 1994; Korpela et al. 1993; Rosenkranz et al. 2010). Mite population growth is slow in colonies established from packages because brood production (and mite reproduction) is limited by the size of the adult worker population. The packages in our study had about 9,000 adult bees and this population declined for about 4 weeks until new adult bees emerged. Predictions from our model on mite population growth in year-1 after colony establishment are similar to those of other models (e.g., Wilkinson and Smith 2002; Vetharaniam 2012). Thus, the high mite levels in the fall were unexpected.

There are several possible explanation that could explain the differences between actual and predicted mite numbers we detected in the fall. We might have underestimated the initial mite numbers in the colonies when they were established. This would have caused a systematic error in predictions that might not have been realized until late summer and early fall. The sugar shake method we used to measure mite populations might not have dislodged every mite. However, we conducted simulations that achieved the October mite numbers we measured in colonies started from packages. The initial mite numbers in colonies would have had to be about 90× higher than what we measured. Furthermore, if the initial mite numbers were high enough to average more than 15 mites per 100 bees in October (e.g., group 4 of the package bee study), in August the mites per 100 bees would have been much higher than the 1.5 ± 0.3 mites per 100 bees we measured in colonies and predicted with the model. The values predicted by the model in the August count would have to be about 11 mites per 100 bees to reach about 15 mites per 100 bees in October.

Another explanation for the differences between actual and predicted mite numbers in September (colonies started from splits) and October (colonies started from packages) was that factors affecting mite reproductive rates were set too low. We used literature values for mite reproductive success and numbers of mated daughter mites per mother mite invading worker and drone cells. However, mite population growth is extremely sensitive to these values, especially the number of offspring per mother mite. We could achieve mite numbers measured in colonies in October in the simulations if we set mite reproduction to 100 % success in worker and drone cells and added an extra mated mite for each singly infested worker and drone cell. However, estimates of mites in colonies in August would have been about 6 mites per 100 bees to reach the October values under the increased reproductive rates. We did not measure such high mite values in any colonies in August.

Mite population growth in colonies started from packages is lower than in established colonies because brood of suitable age for parasitism by varroa is not immediately available. The first cells that become available for parasitism can be multiply infested and this will reduce reproductive rates further (Donze et al. 1996). The model incorporates these constraints on mite reproduction and might have reduced the rate of mite population growth too severely. This might have been particularly important in drone brood since mite reproduction rates are greatest in these cells. In the model, the first drone brood cells that are available for parasitism will have multiple foundresses invade them thus reducing their reproductive rates. If these cells were not multiply infested in the actual colonies, mite reproduction would have been greater than predicted and over time caused the population of mites to increase at a higher rate than predicted.

The increases in mite populations in the fall might have been due to the immigration of varroa from other infested colonies. Others have reported migration of varroa into colonies from foragers with phoretic mites drifting among colonies in apiaries or robbing colonies weakened by high varroa populations (Sakofski et al. 1990; Kraus and Page 1995; Delaplane and Hood 1999; Kralj and Fuchs 2006; Frey et al. 2011). The increase in mite numbers we detected in the late summer and fall was similar to that reported by Sakofski et al. (1990). In that study, varroa migration was low in spring, and then increased considerably during late summer through October. Our colonies were in apiaries that were isolated or near colonies that were treated. Mite migration has been reported to occur from heavily infested colonies that were 1.5 km away (Frey et al. 2011). If immigration of mites occurred in our study, the source would have been from colonies at least 2–3 km away from our study sites.

This study indicates that mite populations in colonies established from package bees and splits can be reduced with HG when colonies are established, and then kept at low levels throughout the summer with additional applications. When there is brood in colonies, three consecutive HG treatments are needed to reduce mite numbers consistently. The rapid growth of mite populations in the fall however, indicates that low mite populations in the summer do not insure that they will remain low as colonies go into the fall and winter. Colonies should be sampled throughout the fall while bees remain active. This study also shows the challenges of maintaining low populations of varroa. Future investigations are needed to quantify immigration rates of varroa throughout the year in commercial apiaries to determine the impact on mite population growth.
